# Articular manifestations in patients with inflammatory bowel diseases treated with anti-TNF

**DOI:** 10.1136/rmdopen-2021-001697

**Published:** 2022-01-28

**Authors:** Laurie Cachen, Gaetane Nocturne, Michael Collins, Antoine Meyer, Aude Gleizes, Salima Hacein-Bey-Abina, Franck Carbonnel, Xavier Mariette, Raphaele Seror

**Affiliations:** 1 Rheumatology, Hospital Bicetre, Le Kremlin-Bicetre, France; 2 Gastroenterology, Hospital Bicetre, Le Kremlin-Bicetre, France; 3 INSERM UMR 996, Faculty of Pharmacy, Paris-Sud University, Paris-Saclay University, Châtenay-Malabry, France; 4 Clinical Immunology Laboratory, AP-HP, Paris-Sud University Hospitals, Le Kremlin Bicêtre Hospital, Le Kremlin-Bicêtre, France; 5 UTCBS, CNRS UMR 8258, INSERM U1022, Faculty of Pharmacy, Paris-Descartes-Sorbonne-Cité University, Paris, France

**Keywords:** tumor necrosis factor inhibitors, spondylitis, ankylosing, antirheumatic agents

## Abstract

**Objective:**

To describe and identify factors associated with articular manifestations occurring in patients treated with anti-tumour necrosis factor (TNF) for inflammatory bowel diseases (IBDs).

**Methods:**

Retrospective monocentric study, including all patients who received an anti-TNF for an IBD in our hospital. All incident articular manifestations occurring during treatment were analysed. Characteristics of patients with paradoxical articular manifestations were compared with that of patients without inflammatory articular manifestations.

**Results:**

Between February 2013 and May 2017, we identified 442 patients (36.2±15 years, 50.5% men) who had ever received an anti-TNF for an IBD: Crohn’s disease (n=277), ulcerative colitis (n=154) and undetermined colitis (n=11). 115 (26%) patients developed new articular manifestations after a mean of 20 (±22) months of treatment. Among them, 59 (13.3%) had inflammatory manifestations: paradoxical in 39%, concomitant of an IBD flare in 27%, linked to an immunisation against anti-TNF in 27% and 7% to another diagnosis. Among paradoxical articular manifestations, 19 (83%) were new articular symptoms, including 8 (35%) de novo spondyloarthritis. There were no predictive factors of paradoxical articular manifestation. Paradoxical manifestations spontaneously resolved in 16 (70%) patients despite continuation of anti-TNF.

**Conclusion:**

Inflammatory articular manifestations occurred in about 13% of patients treated with anti-TNF for IBD. More than a quarter were linked to an immunisation against anti-TNF, which has to be searched in this situation. About 40% were paradoxical. In most of cases, they were transitory and did not require anti-TNFs discontinuation.

**Key messages**

**What is already known about this subject?**

- Paradoxical articular manifestations in patients treated with anti-tumour necrosis factor (TNF) have been described but their frequency and type remain little known.

**What does this study add?**

- Inflammatory articular manifestations occurred in about 13% of patients with inflammatory bowel disease treated with anti-TNF. Only 5.6% of patients had paradoxical articular manifestations, without predictive associated factors found and did not required anti TNF discontinuation most of the time. One quarter was linked to an immunisation.

**How might this impact on clinical practice or further developments?**

- Importance of systematic rheumatologist evaluation in patients treated with anti-TNF for an IBD developing articular manifestation. Immunisation must be systematically searched in the context of new articular manifestation occurring with anti-TNF treatment.

## Introduction

Inflammatory bowel diseases (IBDs) are frequently associated with extradigestive manifestations. Articular manifestations are the most common of them, observed in about 30% of patients.^1 2^ They can be classified in different patterns: axial spondyloarthritis (SpA), peripheral SpA (arthritis or enthesitis or dactylitis associated with IBD), according to the Assessment of SpondyloArthritis International Society (ASAS) criteria^3 4^ or peripheral arthralgia.

Tumour necrosis factor-α (TNFα) is a proinflammatory cytokine that plays a key role in inflammatory process. Anti-TNF monoclonal antibodies were first shown to be effective in chronic inflammatory rheumatic diseases, such as rheumatoid arthritis, psoriatic arthritis or SpA, and then in IBD and their extradigestive manifestations, since the 2000s.^5^ Even if there is evidence supporting the overall good tolerance and safety of anti-TNF biopharmaceuticals, some paradoxical various inflammatory manifestations have been described since their arrival on the market.^6^

Manifestations considered as paradoxical are manifestations for which anti-TNF were shown to be effective and are usually used to treat them. The most common of these manifestations is new occurrence or worsening of psoriasis occurring in around 2%–5% of IBD treated with anti-TNF.^7 8^ But other paradoxical manifestations have been described like uveitis, sarcoidosis.^9 10^ Paradoxical IBD have also been described in patients treated for rheumatic diseases.^11^

Paradoxical articular manifestations occurring with anti-TNF have been less described, and their prevalence and pathophysiology remain unknown. They can be defined as articular manifestations occurring in patients treated with anti-TNF, while IBD is controlled.^12^

The aim of this study was to describe musculoskeletal manifestations in patients with IBD treated by anti-TNF, and to identify their potential risk factors.

## Patients and methods

### Patients

In this retrospective, observational, monocentric study, patients were identified through a systematic research in the electronic database of all patient’s files of Gastroenterology department of Bicêtre, part of AP-HP Université Paris-Saclay Hospitals. Since February 2013, medical files are fully electronic. We identified patients with Crohn disease (CD), ulcerative colitis (UC) or indeterminate colitis (IC) using ICD (International Classification of Diseases)-10 codes: ‘Crohn’s disease’ (K50), ‘ulcerative colitis’ (K51) and ‘other and unspecified non-infective gastroenteritis and colitis’ (K52) whose chart contained any of the following keywords: ‘anti-TNF’, ‘infliximab’, ‘adalimumab’, ‘certolizumab’ or ‘golimumab’ between February 2013 and June 2017. The diagnoses of CD, UC or IC were made by clinicians based on a combination of clinical, biochemical, endoscopic, cross-sectional imaging and histological investigations because, as stated in the most recent IBD guidelines from the European Crohn’s and colitis Organisation, a single reference for diagnosis does not exist.^13^

We excluded patients if they were under 18 years old, had other colitis than CD, UC and IC (ie, infectious, postradiation, postmedication) had never received any therapy with anti-TNF or received it for another diagnosis, had no follow-up after anti-TNF initiation, or if they were not treated in our hospital during anti-TNF treatment without possibility to get access to the file. We analysed patient’s follow-up until October 2018 to have hindsight from anti-TNFs beginning.

All patients of our institution are informed that their clinical data can be used for research and give their consent for the use of their data unless they provide an opposition to it. None of the patients of this study provided an opposition to the use of their data.

### Definition of articular manifestations

For all included patients, medical files were analysed by three rheumatologists (LC, GN and RS) to search for musculoskeletal manifestations during anti-TNF treatment. Manifestations were either persistent stable manifestations or new articular manifestations (including recurrent manifestations after an initial period of remission with anti-TNF), classified as mechanical or inflammatory based on clinical characteristics and/or laboratory and imaging results. Patients with new inflammatory articular manifestations were classified into four groups: (1) articular manifestations evolving in parallel to IBD activity; (2) articular manifestations associated to anti-TNF immunisation; (3) paradoxical articular manifestations (ie, new articular manifestations while IBD was controlled without immunisation) and (4) articular manifestations of other origin.

### Data collection

At anti-TNF therapy initiation, meaning initiation of the first anti-TNF for which the follow-up was available, the following variables were collected: age, sex, type of IBD, disease duration, psoriasis or uveitis history, previous articular manifestations, HLA B27 status when available, treatments received before anti-TNF and associated treatments. For anti-TNF treatment we collected: the first anti-TNF received, the number of line, the type of anti-TNF received, the duration of each anti-TNF and the presence of an eventual immunisation against the anti-TNF defined as antidrug antibodies (ADAs) superior to 10 ng/mL for infliximab and adalimumab and to 5 ng/mL for certolizumab and golimumab.

At the time of the articular manifestation we recorded: the type of manifestation, IBD’s activity assessed by clinical scores (Harvey Bradshow score for CD and partial Mayo Clinic score for UC,^14 15^ comedications, HLA B27 status (if available), C reactive protein (CRP) level, serum anti-TNF trough level and ADA titers. The IBD’s remission was defined, as an Harvey Bradshow score <4 for CD or a partial Mayo clinic score ≤2 for UC, without corticosteroids. CRP could not be used as an IBD’s activity marker, because it could also be increased due to articular inflammatory manifestations. Faecal calprotectin and endoscopic evaluation were not used for assessment of IBD’s activity because they were not systematically done at the time of the articular manifestation.

Then, management and outcome of articular manifestations were analysed.

### Statistical analysis

Descriptive analyses were reported as number with percentage (%) for qualitative variables and as mean±SD or median with IQR, depending on the size population for quantitative variables. To look for factors associated with occurrence of paradoxical articular manifestations, patient’s characteristics were compared between those with paradoxical articular manifestations and those without inflammatory manifestation during anti-TNF treatment (including those with mechanicals manifestations). These comparisons involved non-parametric tests: Fischer’s exact test for qualitative variables and Mann-Whitney U test for quantitative variables. For all statistical analyses, a p<0.05 was considered statistically significant.

## Results

### Patient’s characteristics

Between February 2013 and June 2017, 442 patients with IBD had received an anti-TNF during their follow-up in our centre (table 1). Seventy-four (16.7%) patients had a history of inflammatory articular manifestations before anti-TNF’s initiation: SpA in 37 (8.3%), including 17 (46%) axial, 14 (38%) peripheral and 6 (16%) axial and peripheral SpA, peripheral arthralgia associated with IBD symptoms in 27 (6.1%), and inflammatory manifestations linked to another diagnosis in 10 (2.3%), including eight inflammatory spontaneously regressive arthralgia without specific diagnosis, 1 rheumatoid arthritis and one patient with gout. Nine (2%) patients had history of psoriasis and four (0.9%) patients had history of uveitis before anti-TNF initiation.

**Table 1:** Patients characteristics at baseline

	n=442 (%)
Male gender, n (%)	223 (50.4)
Female gender, n (%)	219 (49.6)
Age (years), mean (SD)	36.2 (15)
Inflammatory bowel disease (IBD)	
Crohn disease, n (%)	277 (62.7)
Ulcerative colitis, n (%)	151 (34.8)
Indeterminate colitis, n (%)	11 (2.5)
Disease duration (years), mean (SD)	6.4 (8.2)
Previous inflammatory articular manifestations, n (%)	74 (16.7)
Peripheral arthralgia associated with active IBD, n (%)	27 (6.1)
Spondyloarthritis, n (%)	37 (8.3)
Others, n (%)	10 (2.3)
Psoriasis, n (%)	9 (2)
Uveitis, n (%)	4 (0.9)
Immunosuppressive therapy before or at the beginning of anti-TNFa	
Azathioprine, n (%)	333 (75.3)
Methotrexate, n (%)	51 (11.5)
Purinethol, n (%)	45 (10.2)
Salazopyrine n (%)	16 (3.6)
Cyclosporine, n (%)	9 (2)
Others, n (%)	17 (3.8)
First anti-TNFa received:	
Infliximab, n (%)	283 (64)
Adalimumab, n (%)	143 (32.4)
Golimumab, n (%)	16 (3.6)
Associated treatment at first anti-TNFa initiation:	
Immunosuppressive therapy, n (%)	265 (60)
Azathioprine, n (%)	214 (48.4)
Purinethol, n (%)	25 (5.7)
Methotrexate, n (%)	24 (5.4)
Others, n (%)	2 (0.5)
Corticosteroïds, n (%)	170 (38.5)
Dose (mg), mean (SD)	37.9 (19.4)

TNF, tumour necrosis factor.

The first anti-TNF received to treat the IBD was infliximab in 64%, adalimumab in 32.4% and golimumab in 3.6%. The mean number of anti-TNF received by patients was 1.4±0.7. During their follow-up, 79.4% of patients received Infliximab, 53.2% adalimumab, 10.2% golimumab and 3.4% certolizumab. Moreover, 0.5% of patients had received etanercept for an associated SpA before IBD’s diagnosis. The mean follow-up of patients was 5.3±2.3 years.

### Frequency of articular manifestations during anti-TNF treatment

**Figure 1:**
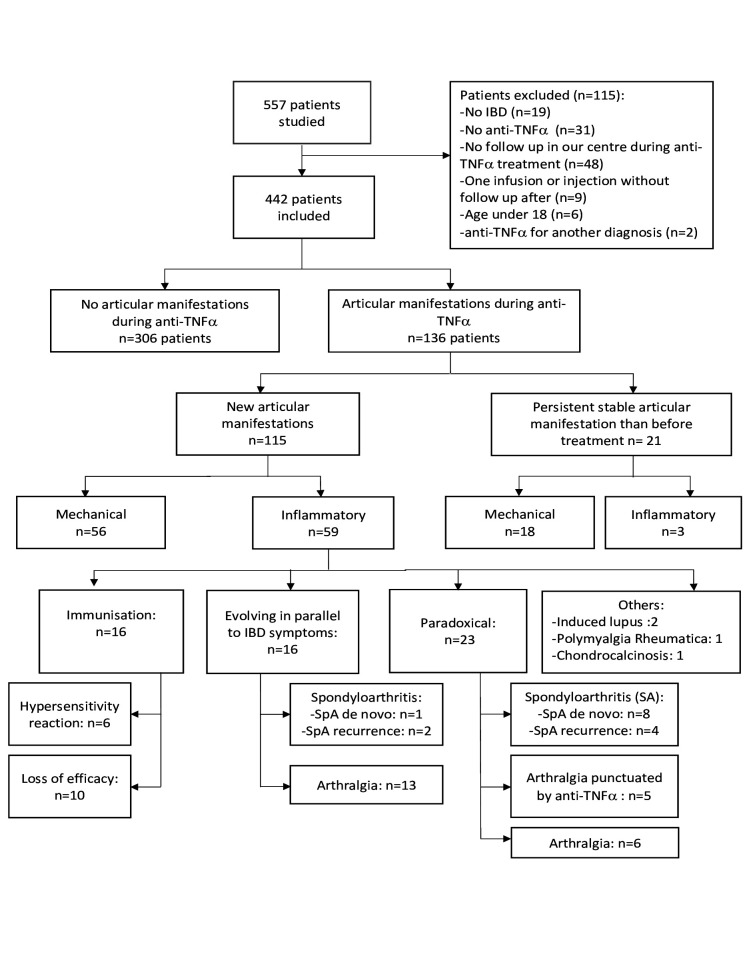
Flow chart. IBD, inflammatory bowel disease; TNF, tumour necrosis factor.

After a mean duration of anti-TNF of 20.5±22.1 months, 136 (30.8%) patients presented an articular manifestation (figure 1).

Among them, 21 (4.8%) had persistent stable articular manifestations that were present before treatment: 18 were mechanical and 3 were inflammatory manifestations. These included one patient with axial SpA and two patients with arthralgia concomitant of IBD flare without improvement with anti-TNF.

The remaining 115 (26%) patients had new articular manifestations: 56 (12.7%) were mechanical and 59 (13.3%) were inflammatory manifestations. Among inflammatory manifestations, 16 (27%) were concomitant of an IBD flare, 16 (27%) were associated to an immunisation against the anti-TNF, 23 (39%) were paradoxical and 4 (7%) were linked to another diagnosis.

### Description and outcome of new articular inflammatory manifestations

#### Articular manifestations evolving in parallel to IBD

During the treatment with anti-TNF, 16 (3.6%) had articular symptoms evolving concomitantly of IBD activity: 13 suffered from peripheral arthralgia, 2 had a flare of an already known SpA and one had a new onset of axial and peripheral SpA. Among them, five improved spontaneously, including four who had early articular manifestations during the first 3 months of treatment. Among the 11 remaining patients, 6 had an optimisation of their anti-TNF treatment (increased injection dose or shortening of between-injection interval), 2 patients had a short corticosteroid therapy, one had addition of Methotrexate and one had a switch for Vedolizumab. For the remaining patient an induced lupus was initially suspected in presence of polyarthralgia with positive anti-DNA. Infliximab was switched to adalimumab and hydroxychloroquine was introduced. But, symptoms persisted and inflammatory back pain and dactylitis occurred. The diagnosis of a new onset SpA was made and a treatment with golimumab was introduced. Overall, evolution was favourable in less than 3 months for 81% of patients.

#### Articular manifestations associated to anti-TNF immunisation

Articular manifestations linked to an immunisation against anti-TNF were observed in 16 (3.6%) patients,14 (87.5%) were treated with IFX and 2 (12.5%) with ADA, after a mean treatment’s duration of 11±10.2 months. It occurred in 4% of all the patients treated with Infliximab and 0.9% of all the patients treated with Adalimumab. Among them, six had hypersensitivity reaction after infliximab infusion including five with delayed hypersensitivity reaction characterised by diffuse peripheral polyarthralgia, myalgia and/or fever, occurring during hours or days after Infliximab administration. The sixth patient had an anaphylactic reaction during perfusion followed 2 days after by polyarthritis. Three of them had high levels of ADA and low anti-TNFα trough levels. For the three other patients, ADA dosage was not available but symptoms were considered as typical. All patients had already been exposed to anti-TNF. For five patients, reaction occurred at the time of reintroduction of infliximab after a long stop. For one patient symptoms occurred at the second Infliximab infusion. Symptoms improved after discontinuation of the anti-TNF in six patients (including five patients who were switching for another anti-TNF), only one required addition of oral corticosteroids.

Among the remaining 10 patients, articular manifestations were due to loss of efficacy induced, for all, by the development of ADA. Three had a flare of a known SpA with symptoms similar to their previous SpA manifestations (including two with concomitant IBD flare), five had inflammatory arthralgia concomitant of an IBD flare and two patients presented their first articular manifestations without IBD’s symptoms. Anti-TNF was stopped in eight patients, including six patients switched for another anti-TNF, leading to symptom’s improvement, and the two others (one presenting a SpA’s flare and the other articular symptoms concomitant of an IBD’s flare) had addition of methotrexate to anti-TNF with improvement and disappearance of ADA. Only one patient, with new isolated articular symptoms, additionally required corticosteroids.

In the whole cohort, 59 (13.4%) patients had an immunisation against anti-TNF. To be noted, 7.9% of all patients have never had any dosage of ADA. Among all 59 patients with immunisation, at anti-TNF initiation, 20 (34%) were co treated with azathioprine, 10 (16%) with methotrexate and 4 (7%) with purinethol. At the time of the development of ADA, only 9 (15%) were still treated with azathioprine, 8 (14%) with methotrexate and 3 (5%) with purinethol. Among all other patients, at anti-TNF initiation, 194 (51%) were treated with azathioprine, 16 (4%) were treated with methotrexate and 22 (6%) with purinethol.

#### Paradoxical articular manifestations

Overall, 23 patients (5.2%) presented paradoxical articular inflammatory symptoms (table 2), including 83% of new articular manifestations and 17% of recurrent ones.

**Table 2:** Characteristics of patients with paradoxical articular manifestation

	n=23
Female gender, n (%)	14 (60.8)
Male gender, n (%)	9 (39.2)
Age	
Mean (SD)	32.2 (10.6)
Median (Q1; Q3)	30.0 (24.5; 39.0)
IBD type	
Crohn disease, n (%)	14 (60.9)
Ulcerative colitis, n (%)	9 (39.1)
Disease duration (years)	
Mean (SD)	5.3 (0.8)
Median (Q1; Q3)	0 (0; 5.5)
Previous articular manifestations, n (%)	6 (26,1)
Spondyloarthritis, n (%)	5 (21.7)
Arthralgia concomitant of IBD’s flare, n (%)	1 (4.3)
Anti-TNFa at the time of paradoxical articular manifestation:	
Infliximab, n (%)	18 (78.3)
Adalimumab, n (%)	5 (21.7)
Associated immunosuppressive treatment	8 (34.7)
Methotrexate, n (%)	1 (4.3)
Azathioprine, n (%)	5 (21.7)
Purinethol, n (%)	2 (8.7)
Anti-TNFa’s line:	
First, n (%)	19 (82.7)
Second, n (%)	3 (13)
Third, n (%)	1 (4.3)
Time between anti-TNFa’s initiation and occurrence of articular manifestation:	
Mean (SD)	29.4 (13.4)
Median (Q1; Q3)	24.0 (10.5; 34.0)
Paradoxical articular manifestation’s characteristics:	
Peripheral, n (%)	18 (78.2)
Arthralgia, n (%)	13 (56.4)
Arthritis, n (%)	5 (21.8)
Enthesitis, n (%)	1 (4.3)
Axial, n (%)	2 (8.7)
Peripheral and/or enthesitis and axial, n (%)	2 (8.7)
HLA B 27:	
Negative, n (%)	1 (4.3)
Unknown, n (%)	22 (95.7)
CRP:	
Unknown, n (%)	1 (4.3)
Mean, mg/L (SD)	11.9 (18.7)
Median, mg/L (Q1; Q3)	7.0 (1.0; 24.0)
Anti-TNFa Abs:	
Negative, n (%)	21 (91.3)
Unknown, n (%)	2 (8.7)
Anti-TNFa dosage	
Mean, mg/mL (SD)	9.5 (7.3)
Median, mg/mL (Q1;Q3)	9.5 (8.8;10.2)
Unknown, n (%)	2 (8.7)
Management:	
Continuation anti-TNFa, n (%)	17 (74)
Addition of another immunosuppressive therapy, n (%)	2 (8.7)
Discontinuation of anti-TNFa, n (%)	4 (17.3)
Switch for another anti-TNFa, n (%)	3 (13.0)
Continuation of immunosuppressive therapy alone, n (%)	1 (4.3)

CRP, C reactive protein; IBD, inflammatory bowel disease; TNF, tumour necrosis factor.

Paradoxical articular manifestation occurred with infliximab in 78% and with adalimumab in 22%, representing 5.1% of all patients who received infliximab, and 2.1% adalimumab. It was the first anti-TNF in 82%. Overall, 35% of patients had another immunosuppressive therapy at the time of the paradoxical articular manifestation. None of these patients were taking corticosteroids. The mean duration between anti-TNF initiation and first articular symptoms was 29.4±13.4 months. The mean duration between anti-TNF initiation and first articular symptoms was 29.4±13.4 months. This delay was not influenced by concomitant immunosuppressive therapy (p=0.27).

Among these 23 patients, 8 had a new onset of SpA (5 peripheral, 2 axial and 1 axial and peripheral SpA), fulfilling ASAS criteria.^3^ To note, three of these eight patients had an associated treatment at the time of the manifestation (two had azathioprine and 1 purinethol). Their management consisted in switching for another anti-TNFα in 2, with improvement for both, addition of methotrexate in 2, with improvement for one and unknown evolution for the other, local corticosteroid injection in one. In three patients, the same anti-TNF was continued with improvement of symptoms. None of them received oral steroids. Four patients had a flare of their known SpA (two axial SpA and two peripheral SpA). Clinical phenotypes of these flares were similar to their previous SpA manifestations. One of them had an associated treatment (Methotrexate) to the anti TNF at the time of the manifestation. Treatment was continued in all. Five patients had peripheral arthralgia following infliximab injection without ADAs. Arthralgia spontaneously improved in four and only one patient had to discontinue treatment and was switched for another anti-TNF, with improvement of symptoms. The remaining six patients had peripheral continuous arthralgia without fulfilling criteria for SpA or other rheumatic disease. One patient had concomitant occurrence of polymorphous erythema, the treatment was discontinued because of the cutaneous manifestations, which lead to articular symptoms improvement. For the five other patients treatment was continued, and symptoms improved spontaneously in less than 3 months.

Of note, among these 23 patients, one patient with articular paradoxical articular manifestation, had also a paradoxical cutaneous psoriasis induced by anti-TNFα, but years later.

We have looked for factors associated to the occurrence of paradoxical articular manifestation by comparing all the 23 patients to all patients without new inflammatory articular manifestation during anti-TNF. We found that the only predictive factor of paradoxical articular manifestation was to have an history of SpA before anti-TNF treatment (table not shown). So, we decided to exclude patients with history of inflammatory articular manifestation before anti-TNF in this analysis, to be sure to not hide another predictive factor (table 3). Among baseline characteristics, no associated factors were found. However, we found a statistical longer duration of anti-TNF for paradoxical articular manifestation. To overcome this bias, we realised an analysis including only patients who had a paradoxical articular manifestation during the two first years of anti-TNF to those without new inflammatory articular manifestation and who had a minimum of two consecutive years of treatment with anti-TNF (online supplemental table S1). There were no factors associated to paradoxical articular manifestation. Sensitivity analysis performed on the subset of patients having real arthritis (n=5) provided similar results. Of note, none of these five patients was taken concomitant disease modifying anti-rheumatic drug (DMARD) in association with anti-TNF at the time of articular manifestations occurrence.

10.1136/rmdopen-2021-001697.supp1 Supplementary data

**Table 3:** Comparison between patients with paradoxical articular manifestation and those without new inflammatory articular manifestation during anti-TNF therapy

	Patients with paradoxical articular manifestation, n= 17 (%)	Patients without new inflammatory articular manifestation, n=322 (%)	P value
Female gender, n (%)	11 (64.7)	142 (44.1)	0.133
Male gender, n (%)	6 (35.3)	180 (55.9)	0.133
Age (years), mean (SD)	31.4 (10.9)	36.2 (15.6)	0.329
Inflammatory bowel disease			
Crohn disease, n (%)	9 (52.9)	195 (60.6)	0.614
Ulcerative colitis, n (%)	8 (47.1)	118 (36.6)	0.443
Indeterminate colitis, n (%)	0 (0)	9 (2.8)	1
Disease duration (years), mean	6.9 (7.6)	5.8 (7.4)	0.658
Psoriasis, n (%)	0 (0)	5 (1.6)	1
Paradoxical psoriasis, n (%)	1 (5.9)	14 (4.3)	0.546
Uveitis, n (%)	0 (0)	1 (0.3)	1
No of anti-TNFa received, mean (SD)	1.5 (0.7)	1.4 (0.6)	0.377
Immunosuppressive therapy at the beginning of anti-TNFa, n (%)			
Azathioprine, n (%)	11 (64.7)	197 (61.2)	1
Methotrexate, n (%)	11 (64.7)	162 (50.3)	0.322
Purinethol, n (%)	0 (0)	12 (3.7)	1
Others, n (%)	0 (0)	21 (6.5)	0.612
Anti TNF Abs	0 (0)	2 (0.6)	1
Positive, n (%)	0 (0)	33 (10.2)	0.39
Follow-up, mean, years (SD)	6.5 (3.3)	5.1 (2.8)	0.063
Total anti-TNFa duration, mean, years (SD)	5.6 (3.0)	3,3 (2.6)	0.00002

TNF, tumour necrosis factor.

#### Articular manifestation linked to other diagnoses

Finally, four patients had other manifestations, including induced lupus (n=2), chondrocalcinosis (n=1) and polymyalgia rheumatica (n=1). Among patients with induced lupus, one, treated with infliximab, had polyarthralgia, tenosynovitis and chilblain lupus, associated with positive antinuclear antibodies (1/640). Infliximab was discontinued and hydroxychloroquine was introduced with complete resolution. The other patient developed, during golimumab treatment, a polyarthritis with skin lesions of leucocytoklastic vasculitis and positive antinuclear antibodies (1/1280). Golimumab was stopped leading to improvement of symptoms.

## Discussion

Our study is one of the largest analysing systematically occurrence of musculoskeletal manifestations in consecutive anti-TNF treated IBD real-life patients. In our cohort, about 30% of the patients presented musculoskeletal manifestations during treatment with anti-TNF, suggesting that it could be a frequent issue. However, only half of those were inflammatory articular manifestations and even less (23 patients (5.2%)) were considered as paradoxical, that is, flare or new onset of articular manifestations whereas IBD was in remission. There were no factors associated to the occurrence of paradoxical articular manifestation. Of note, nearly the same proportion of patients (16 patients (3.6%)) presented articular manifestations linked to immunisation against the anti-TNF.

In the literature, the frequency of paradoxical articular manifestations ranged from 1.6% to 11%.^6 16–18^ These variations could be explained by different methodologies leading to a potential underestimation in retrospective studies and also by different definitions of paradoxical articular manifestations. Indeed, in some studies patients with articular manifestation concomitant of immunisations were considered as paradoxical, whereas we considered them separately and in some other studies patients with previous articular manifestations before anti-TNF’s beginning were excluded. The highest frequency was observed in a prospective study including 80 patients treated with Infliximab for IBD, where paradoxical manifestations were reported in 9 (11%) patients, that is, twice more frequent than in our cohort. These articular manifestations were more frequently peripheral (6/9), which is similar to our study (78%) and only 1/3 patients had SpA as opposed to half in ours. Interestingly, three patients also had paradoxical cutaneous psoriasis, what we did not observe in our cohort. As in our study they did not found any factor associated with the occurrence of paradoxical articular manifestations.

Understanding of paradoxical manifestation pathophysiology has progressed during the last years, especially in paradoxical psoriasis. It has been shown that even if classical and paradoxical psoriasis have clinical similitudes, their pathophysiology differs; indeed, classical psoriasis is a T-cell mediated disease driven by TNF, by contrast paradoxical psoriasis is a type-1 interferon-driven innate inflammation.^19 20^ However, articular paradoxical manifestation pathophysiology remains unknown, some data suggested involvement of IL-12/IL-23 pathway. A recent study of synovial biopsies of 10 patients with paradoxical articular manifestations showed histological findings closer to that of psoriatic arthritis than in seronegative rheumatoid arthritis.^21^ Moreover, in addition to their binding to TNF, infliximab and Adalimumab are able to bind, with their Fc region, to the Fc γ-receptor CD64 and CD16/32 expressed by monocytes/macrophages leading to the paradoxical release of proinflammatory cytokines as IL-12 and IL-23.^22 23^ These findings suggest that inhibition by IL-12/IL-23 inhibitors could be an effective option in patients with disabling paradoxical articular manifestation.^21^

Today there is no recommendation for the management of articular paradoxical manifestations. However, in our study, only 4 (17%) patients with paradoxical articular manifestation had to discontinue anti-TNF, including three who began another anti-TNF without recurrence of articular symptoms. In previous studies, paradoxical events also rarely led to anti-TNF discontinuation.^16–18^

Another interesting finding, is that a similar proportion of patients (3.6%) developed articular manifestations linked to an immunisation against the anti-TNF. Of note, in case of lack of efficacy, 1/3 of patients had only articular symptoms without IBD’s ones supporting the idea that immunisation against anti-TNF must be searched when articular manifestations occurred even in the absence of IBD’s flare. Precedent studies had shown that associated immunosuppressive therapy to anti-TNFα could prevent the development of ADA. This have been largely demonstrated in rheumatoid arthritis with the use of methotrexate,^24^ but in SpA and IBD data are fewer.^25 26^ Here, our study was not powered and designed for that purpose. Contrarily on paradoxical manifestations, in case of immunisation, treatment discontinuation with switch for another biologic is necessary and lead to improvement of symptoms in most of cases.

Even if our study is the first describing, with such a significant number of patients, the occurrence of articular manifestations during anti-TNF treatment, there are some limitations. First, it is a retrospective design, with no systematic examination by a rheumatologist, subject to a reporting bias and some imprecision on description of articular manifestations. Indeed, due to the retrospective nature of the study, we cannot certify that we did not miss some cases of inflammatory articular manifestations for example by misclassing some mechanical manifestations. However, if it is the case these manifestations were minor and not severe since patients were not referred to rheumatologist. Nevertheless, our electronic search ensures to have include all consecutive anti-TNF treated patients during our study period. With this methodology, we have excluded a possible recruitment bias and ensured the exhaustiveness and representativeness of our population. In addition, most of the patients (60%) that presented persistent inflammatory manifestations were seen by a rheumatologist. Also, we were not able to assess the potential impact of some factors, such as HLA-B27, due to missing data. Finally, all patients were recruited in a tertiary university hospital centre, which might have selected more severe patients with IBD. However, in France, patients requiring anti-TNF were, at the time of the study, systematically referred to hospital for anti-TNF initiation.

To conclude, in this real-life study, inflammatory articular manifestations occurred in about 13% of patients treated for IBD with anti-TNF. More than a quarter of them were linked to an immunisation against anti-TNF, with either hypersensitivity reactions or loss of efficacy on articular and/or, but not necessarily, digestive symptoms. Thus, ADAs must be searched in such situation. Finally, only 5.6% of the patients presented paradoxical manifestations. They were frequently transitory and did not require anti-TNF’s discontinuation, with spontaneous improvement. There were no predictive factors of the occurrence of paradoxical articular manifestation in our study.

## Data Availability

All data relevant to the study are included in the article or uploaded as online supplemental information. Raw data coming from electronic medical records cannot be shared.
