# Supplementary material for: Articular manifestations in patients with inflammatory bowel diseases treated with anti-TNF
Source: RMD Open. 2022 Jan 28;8(1):e001697. doi: 10.1136/rmdopen-2021-001697 (PMC8804691; doi:10.1136/rmdopen-2021-001697)
Supplement: Supplementary data [file rmdopen-2021-001697supp001.pdf]

Supplementary Table S1:_Comparison between patients with paradoxical articular manifestation during the first two years of anti-TNF treatment and those without new inflammatory articular manifestation during anti-TNF therapy and who had a minimum of two consecutive years of anti-TNF treatment

	Patients with paradoxical articular manifestation, n= 9 (%)	Patients without new inflammatory articular manifestation, n = 187 (%)	p
Female gender, n (%)	6 (66.7)	77 (41.2)	0.172
Male gender, n (%)	3 (33.3)	110 (58.8)	0.172
Age (years), mean (SD)	31.1 (7.9)	33.4 (11.5)	0.840
Inflammatory bowel disease (IBD)			
Crohn disease (CD), n (%)	6 (66.6)	126 (67.4)	1
Ulcerative Colitis (UC), n (%)	3 (33.3)	57 (30.5)	1
Indeterminate colitis, n (%)	0 (0)	4 (2.1)	1
Disease duration (years), mean	4.8 (4.4)	4.8 (4.8)	0.795
Psoriasis, n (%)	0 (0)	2 (1.1)	1
Paradoxical psoriasis, n (%)	1 (10)	12 (6.4)	0.468
Uveitis, n (%)	0 (0)	1 (0.5)	1
Number of anti-TNFα received, mean (SD)	1.4 (0.6)	1.4 (0.5)	0.919
Immunosuppressive therapy at the beginning of anti-TNFα, n (%)	8 (88.9)	113 (60.4)	0.157
Azathioprine, n (%)	7 (77.8)	94 (50.3)	0.171
Methotrexate, n (%)	0 (0)	7 (3.7)	1
Purinethol, n (%)	1 (11.1)	11 (5.9)	0.441
Others, n (%)	0 (0)	1 (0.5)	1
